# What interdependence can tell us about collaborative learning: a statistical and psychological perspective

**DOI:** 10.1186/s41039-018-0084-x

**Published:** 2018-10-16

**Authors:** Lenka Schnaubert, Daniel Bodemer

**Affiliations:** University of Duisburg-Essen, Lotharstrasse 65, 47057 Duisburg, Germany

**Keywords:** Interdependence, Intra-class correlation, Collaborative learning, Experimental study, Group awareness, Metacognition

## Abstract

When learning collaboratively, learners interact and communicate transactively. Interventions to foster collaborative learning frequently target such interactive processes and thus may drastically change how learners engage with and thus influence each other. One statistical phenomenon related to collaborative learning is the interdependence of data gained from learners collaborating. Often viewed as a mere statistical phenomenon, on a conceptual level, statistical interdependence is a similarity between learners mainly resulting from the mutual influence learners have on each other while collaborating and is thus closely related to collaborative practices. In this paper, we report data of an exemplary study (*N* = 82) to illustrate how information on interdependence and within- and between-dyad variance may add to data interpretation. The study examined how providing metacognitive group awareness information during collaboration affects individual learning outcomes. We found indications that the information fosters knowledge gain, but not confidence. Surprisingly, the data revealed different levels of interdependence between conditions, which led us to assume interdependence to be part of the treatment effect resulting from differential collaboration processes. We discuss reasons and implications of varying levels of statistical interdependence and their impact on inferential and descriptive statistics.

## Introduction

Collaborative learning (CL) yields a lot of potential to foster knowledge construction. When learning collaboratively, learners interact and communicate transactively. They exchange and commonly build knowledge and/or skills. Interventions to foster collaborative learning frequently target such interactive processes and thus may drastically change how the learners engage with and thus influence each other. However, research on collaborative learning comes with a number of additional challenges. One important issue is that collaboration is an interactive activity of learners that is thought to foster not only group performance but also individual learning (Hesse 2007). Thus, the data collected is frequently on different levels (individual and group) and/or heavily intertwined (like in turn-taking during discussion) (cf. Strijbos and Fischer 2007). This poses a great challenge for quantitative research, because traditional analyses (like ANOVAS) require independent data and are not designed to handle statistical interdependence (cf. Janssen et al. 2011).

While there have been promising developments like multi-level approaches to deal with hierarchical data, these approaches are often limited especially when working with dyadic data and/or require high standards like large sample sizes (Janssen et al. 2011; Nezlek et al. 2006). Thus, dealing with hierarchical data usually comprises of testing for interdependence using for example the intra-class correlation (ICC) before deciding on an appropriate strategy. If the ICC indicates practically relevant levels of interdependence, the data is analyzed accordingly by accounting for the non-independence (Cress 2008). There are different ways to handle interdependence (cf. Janssen et al. 2011). For example, data is sometimes analyzed on group level loosing information about individual data; however, this approach may have downsides (e.g., increasing the risk of type 2 errors by losing statistical power due to reduced sample size). Recently, more and more researchers use multi-level approaches to account for interdependence. However, dyadic data provides a special challenge for the later, since the usual regression-based approaches are not appropriate (Kenny and Kashy 2011). If the data shows no signs of interdependence, statistically, the dyads do not have to be taken into consideration and independence may be assumed. While this often solves analytical problems, theoretically, this may be a short-sighted perspective. To get to the bottom of this, we first need to take a closer look at what statistical interdependence means statistically, but also conceptually.

Statistically, interdependence means that data (for example of specific subjects within a sample) is correlated; in CL research, this is usually measured with the intra-class correlation coefficient (ICC) (Griffin and Gonzalez 1995; Kenny et al. 2006; Shrout and Fleiss 1979). The ICC measures the percentage of variance due to belonging to the same group or dyad. One interpretation is thus, how much of the variance between subjects may be explained by the (random) dyad factor (Gonzalez and Griffin 2012; Griffin and Gonzalez 1995; Kenny et al. 2006). Thus, the more similar members of a dyad are (in comparison to members of the whole sample), the higher the value. Conceptually, positive statistical interdependence resulting from collaboration describes a similarity between learners that had been in the same group or dyad during collaboration.

To grasp the theoretical/psychological meaning of statistical interdependence within CL research, it is important to take a look at how interdependence occurs within collaborative learning scenarios. Statistical interdependence of learners after collaboration may have various causes. According to Cress (2008), assuming random assignments to a group or dyad (no compositional effects), these causes are common fate and reciprocal influence. Common fate refers to unique experiences learners in a group share during collaboration by being confronted with the same influences within the learning environment, e.g., when following the same discussion thread or listening to the same arguments. Taking this further, within CL, learners are supposed to actively interact and thus influence each other; this is known as reciprocal influence. Thus, learners influence each other’s cognitions, motivation, and behavior, which may lead to both greater differences between groups and convergence within the groups. Within CL research, reciprocal influence is important not only because it is the main cause of interdependence (Bonito 2002; Cress 2008), but because it is the core of collaboration. However, interdependence is not only a phenomenon observed within collaborative learning processes but also within individual learning outcomes that rely on collaboration processes. Depending on the outcomes measured, results of collaborative efforts may be heavily interdependent between learners in a group or dyad due to mutual influence caused by interactive processes. While these processes do not have to cause statistical interdependence that is visible in the data a researcher is interested in, conceptually, interrelations should be expected for variables directly related to collaborative practices. Especially learning outcomes like knowledge are expected to be highly impacted by knowledge exchange and mutual knowledge building processes as they highly depend on interactive processes.

For example, imagine a scenario with all learners having unique prior knowledge on a subject and being brought together in dyads in an environment designed to share their knowledge and build a common knowledge base. The learning partners then start exchanging their knowledge by externalizing internal cognitive information for the group’s benefit and by perceiving observable group level activities. Ideally, they each share (i.e., externalize) all relevant content information they possess on the subject, while processing, comprehending, and elaborating on the information (Buder 2017). Further, they might detect misconceptions and correct them together. In this scenario, the content of their knowledge after collaboration would be rather similar due to convergence (Weinberger et al. 2007). Additionally, their levels of knowledge would be interdependent since the amount of shared information contributes to the amount of knowledge they have on the subject. Moreover, if we assume reciprocal processes, collaboration should benefit both learners similarly, albeit not identically. For example, high-quality collaboration should benefit both learners, while poor strategic decisions should hamper learning for both. Of course, going beyond knowledge exchange and toward collaborative knowledge building activities, the relation between the collaborative activities and interdependence of outcomes are even more pronounced, since these processes rely on transactivity (cf. Teasley 1997). Obviously, there are other scenarios in which learning processes are not balanced, but for example complementary or unilateral. In these scenarios where the collaborative processes lack reciprocity, learners may profit very differently. However, even within less reciprocal settings, learners may influence each other in a unique way that fosters interdependence: a weak learner might for example profit from a more knowledgeable partner not only by internalizing the information given by the partner but also by adopting the other one’s thought processes. In such a scenario, learning outcomes may be highly interdependent even though learner partners profit from collaboration very differently.

Even though the mechanisms behind interdependent data may highly depend on the concept measured and on the processes expected within collaboration, whenever inter- or even transactive learning activities occur, a certain degree of interdependence can be expected in outcomes directly related to collaborative practices. Thus, although not definitive, a lack of interdependence may indicate an undesirable lack of such collaborative processes. A lack of interdependence may indicate problems with the theoretical assumptions about collaborative practices and/or with the actual collaboration processes happening within the experimental design and should be critically addressed. Although some researchers point out that interdependence should be studied and not merely eliminated (e.g., Gonzalez and Griffin 2012) and reciprocal influence—a major cause of statistical interdependence—is desired within CL (Cress 2008), a lack of statistical interdependence in the data after collaboration is seldom commented on within CL research, let alone discussed in detail.

Altogether, this means that interdependence is indicative for collaborative processes (which does not mean that the absence likewise is inevitably and indicator of the lack of such) and should thus be celebrated rather than bemoaned. It also means that the collaborative processes taking place strongly influence the level of interdependence between learners within a dyad or group. This is important, since interventions targeting these processes may not only have an impact on these collaborative learning processes and individual learning outcomes but also on the relatedness/interdependence of these outcomes. Every time interventions targeting collaboration processes are believed to affect individual learning outcomes, they may affect the level of interdependence of these outcomes as well, especially if they are specifically designed to foster individual knowledge construction via such mechanisms. Thus, statistical interdependence of data of learners within dyads may not always be similar between experimental conditions within an experimental setup.

If we connect information about the nature of psychological research and treatment effects on interdependence with statistical practices, it is surprising that while statistically straightforward, in practice, interdependence is usually measured on the whole sample. Apart from ignoring possible differences in interdependencies between experimental conditions, this additionally conflates treatment variance with variance due to dyad (interdependence of learners within a dyad will be inflated in cases of between-dyad variations of treatments, especially when effects are large, and deflated for within-dyad variations). While one can correct for this effect by factoring out treatment variance (and only use dyad and residual variance to estimate interdependence), this is seldom explicitly reported. And even if the treatment effect is adjusted for, such a procedure still assumes that the variance due to belonging to the same dyad is comparable between experimental conditions. Conceptually, this is a bold and even flawed assumption considering that interventions varied within an experiment often target interactive processes within the collaborative situation.

To sum it up, in this paper, we argue that (1) statistical interdependence after collaboration is something to be expected and even hoped for in CL; (2) assessing the ICC on a sample level is flawed on principle, because variance caused by the treatment will taint the results and lead to overestimations of interdependence within dyads; (3) interdependence can be highly influenced by interventions targeting collaborative learning processes and may thus differ dramatically between experimental conditions; and (4) information on interdependence is valuable and indicative of collaborative processes, and thus should be explicitly and critically reviewed. To illustrate this, we will describe data of an exemplary study to show how a treatment designed to foster interactive processes between individuals learning in a dyadic setting may affect interdependence, which in turn affects the data assessed. While we are aware that multi-level approaches may account for such differences, we argue that statistical interdependence is not primarily a statistical nuisance to be eliminated from our data, but a valid diagnostic outcome to be explicitly discussed in research on collaborative learning, as it is the core of collaboration. The dataset we present is drawn from a study designed to investigate how metacognitive information in group awareness tools affects collaborative learning outcomes in a dyadic setting. Due to the methodological focus of this paper, we will only briefly sketch the theoretical background, research questions, and methods of this exemplary study. We will then describe the results of statistical analyses by comparing individual and dyadic data in detail and discuss the results with a specific focus on the value of information on interdependence.

### Interdependence in CSCL: an example from group awareness research

In many studies within computer-supported collaborative learning research (CSCL), interventions are thought to foster collaboration processes that—in turn—foster individual processes (often cognitive in nature) leading to better knowledge acquisition or skill development. Progress in ICT makes it possible to support these collaborative learning processes in various ways. One typical example is group awareness tools. Group awareness tools are specifically designed to inform learners about cognitive, social, and/or behavioral aspects of group members or the group as a whole in order to implicitly guide their learning processes to ultimately benefit individual learning (Bodemer et al. 2018). By providing relevant information without giving an explicit structure or instructions, this approach builds heavily on individual skills and therefore enables diverse approaches to learning. While tools providing (cognitive) group awareness information can support relevant learning processes (cf. Janssen and Bodemer 2013), empirical research uses a great variety of target concepts, some of which may well be framed within a metacognitive context (e.g., Dehler et al. 2011). However, the field lacks a thorough investigation of the role of metacognitive awareness information in collaborative learning. Thus, in our experimental study, we aim to investigate whether metacognitive information has an added bonus to mere cognitive content information for both cognitive and metacognitive learning outcomes, drawing on group awareness research on collaborative learning and metacognitive research on individual self-regulation. While analyzing the data, we will look in detail at interdependencies between learners and compare individual and dyadic approaches to data analyzes.

### Metacognitive group awareness information: research questions and hypotheses

Group awareness tools foster collaborative learning processes by providing learners with relevant information about other learners within their group or the whole group in order to make them aware of their individual or common status (Bodemer and Dehler 2011). For example, they may visualize individual needs or conflicting opinions or assumptions (Engelmann et al. 2009), helping learners to identify what aspects of the learning material need further attention. Thus, learners may use the information to structure their common learning processes. Additionally, providing social context information may foster grounding processes and partner modeling. These processes are vital for effective collaboration (Dillenbourg 1999), because they may help learners to coordinate their common learning processes (Clark and Brennan 1991) and to tail their conversation to the specific needs of the individuals (Clark and Murphy 1982) to foster effective communication. Ultimately, this is assumed to foster knowledge exchange processes and constructing shared knowledge. Empirically, such tools have shown to foster individual knowledge gains as well (e.g., Bodemer 2011; Sangin et al. 2011).

However, there is a multitude of tools providing very different kinds of information assessed in very different ways (for an overview on social and cognitive group awareness tools, see Janssen and Bodemer 2013). For example, some tools provide information about the content of other learners’ cognitions, fostering awareness about conflicting assumptions within the group (e.g., Bodemer 2011), while others provide more contextual information (cf. Engelmann et al. 2009). From a metacognitive perspective, the latter tools may provide information on learners’ metacognitive self-evaluations rather than cognitions (e.g., Dehler et al. 2011). Subjective evaluations of knowledge have an inherent stand-alone value (Efklides 2008) exploited by group awareness research: they indicate subjective needs by pointing out uncertainties or lacks of knowledge (Engelmann et al. 2009). Utilizing such metacognitive information is part of the causal chain for successfully self-regulating learning (Nelson et al. 1994). Additionally, metacognitions may validate cognitive information by giving a subjective value to objectively evaluable assumptions. For example, confidence in response ratings (usually seen as basic metacognitive judgments, cf. Dunlosky and Metcalfe 2009) may be seen as giving value to otherwise meaningless responses or assumptions: without a high degree of subjective certainty attached to assumptions, these may not be viewed as knowledge and may not guide real-life decisions and behavior (cf. Hunt 2003). While information on contents of knowledge can foster awareness about socio-cognitive conflicts and thus coordination efforts of the learning process, additional confidence information might change how such conflicts are handled (Schnaubert and Bodemer 2016). Ultimately, metacognitive confidence information may provide social context information that may help learners interpret their partners’ knowledge and their communication efforts.

Through the above described mechanisms, confidence information may lead to better aligned communication and help learners to better ascertain knowledge distributions and control/adapt knowledge exchange processes. While this should foster knowledge gain, knowledge about learning partners’ confidence in assumptions may also enable to interpret the assumptions themselves in terms of conflict perception and may thus help resolve these conflicts more efficiently. We already established that fostering knowledge exchange processes through intervention may also impact knowledge interdependence. It follows, then, that higher knowledge interdependence with metacognitive confidence information could be expected.

Confidence information may also impact the interdependence of confidence levels within groups. For example, confidence might be an indicator for successfully resolving uncertainties or epistemic conflicts. Since these resolution processes are at least similar for learners within a dyad (cognitive processes may differ, but the arguments and interactive processes the learners are exposed to are the same), the outcomes are also expected to be somewhat aligned. But even without active interaction, confidence within dyads may be aligned. Metacognition research within the area of social influence and social consensus has found that being confronted with social information may make learners start doubting their own estimations if there is a mismatch between the social information (e.g., performance) and their own estimation of item difficulty (Fraundorf and Benjamin 2016). Similarly, consensus in a group fosters certainty and being aware of controversies may be detrimental to individual confidence levels (e.g., Luus and Wells 1994; Yaniv et al. 2009). Since these concepts (consensus and controversy) both emerge on group level, they should apply similarly to members of the same dyad, but not members of different dyads, thus fostering interdependence.

In sum, metacognitive information may support learners in identifying aspects of the learning material that need further attention. Additionally, they may foster grounding processes enabling learners to tail their conversation and learning process better to the needs of the individuals and thus should foster knowledge gain. Consequently, we assume that learners provided with metacognitive group awareness information in the form of confidence regarding specific assumptions gain more knowledge during collaboration than learners without this information (hypothesis 1) due to improved collaboration processes. However, we acknowledge the possibility that additional information might also put an extra strain on the learners’ cognitive system, already charged by the collaborative situation (Dillenbourg and Bétrancourt 2006). On the other hand, metacognitive confidence information may also directly affect learners’ confidence levels, since insecurities signaling an individual need for clarification can easily be identified and thus addressed during collaboration—leading to a higher clear-up rate. For example, Dehler et al. (2011) found that providing information on self-assessed levels of understanding led learners to tailor their communication to these aspects. Thus, we further hypothesize that learners receiving information on metacognitive confidence regarding specific assumptions gain more confidence during collaboration than learners without this information (hypothesis 2). In terms of interdependence, we expect enhanced knowledge exchange and conflict resolution processes to increase interdependence between learners within the same dyad.

## Methods

To answer our research questions concerning the influence of metacognitive awareness information on collaborative learning and to study the impact of such an intervention on the structure (i.e., interdependence) of the data assessed, we evaluated data of an experimental study with 41 dyads of learners (82 subjects), randomly assigned to the (between-dyad) research conditions. They were all university students (55 female, 27 male) with ages ranging from 18 to 31 years (*M* = 22.01, *SD* = 3.13). Dyads were uni- and mixed sex. The study was conducted in accordance to the ethics guidelines of the German Psychological Society and approved by the ethics committee of the university. All participants gave their explicit and informed consent. We focus our analyses on one between-dyad factor varying the availability of metacognitive confidence information (MC) during collaboration. Consequently, we will compare two research conditions: one only receiving information on the learners’ assumptions during learning (MC−) and one additionally receiving information on the learners’ metacognitive confidence with regard to the assumptions (MC+). We then measure how collaboration affects learning outcomes by measuring the data pre and post collaboration (within-subject factor time). However, the complete design included another between-dyad factor (availability of information on overall pre-test performance), making it originally a 2 × 2 × 2 design with repeated measures on one factor, counterbalanced regarding factor levels. Since it is not the focus of the current paper and the factors did not interact in influencing any of the dependent variables (multivariate interaction for both between dyad factors and the within-dyad factor time: *F*(2, 77) = 0.07, *p* = .935, η_p_^2^ < .01), we limit our analyses to the first factor (metacognitive confidence information). Thus, the data set provides a typical example of research on collaborative learning in which a treatment is implemented on dyad level to foster beneficial collaboration of learning partners interacting in a dyadic setting, and individual outcome measures are measured pre and post collaboration.

### Procedure

**Fig. 1:**
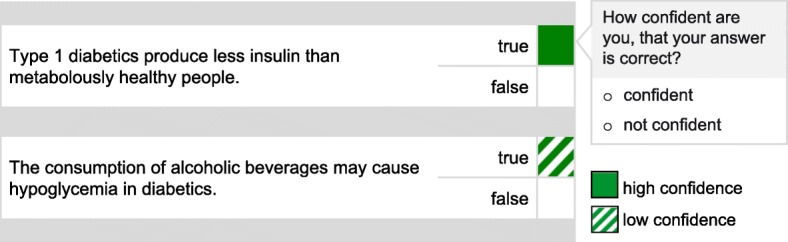
Examples of translated tasks with answers and confidence rating

All experiments were conducted in our research lab with learners working individually on a computer and in dyads on a multi-touch tabletop. Again, this is quite common in CSCL studies, where typically individual and collaborative parts of the experiment alternate. After welcoming the participants, two learners were simultaneously placed in front of a computer screen each and started the experiment individually. After filling out questionnaires, e.g., about demographics, each learner received a text on diabetes mellitus and blood-sugar regulation and had up to 15 min to study the text. In order to foster within-dyad knowledge interdependence and support interactive engagement in the task (cf. Deiglmayr and Schalk 2015), each learner in a dyad received different text versions, that shared basic information on blood-sugar regulation available to both learners, but had a different focus especially on diabetes mellitus. They then each individually answered 18 binary true-false questions about the content of both texts and provided binary confidence ratings on each item. Answers given with confidence were visualized green, unconfident answers were visualized hatched white-green (cf. Fig. 1).

**Fig. 2:**
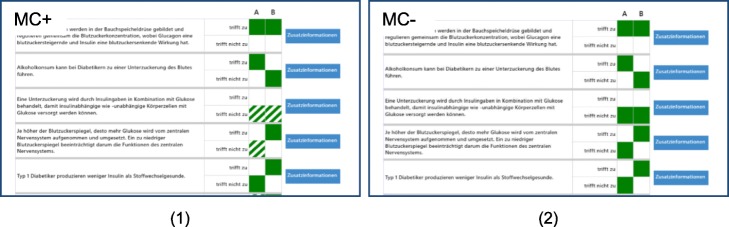
Arrangement of collaborative learning scenario on the multi-touch tabletop; (1) with metacognitive information available (MC+); (2) without metacognitive information available (MC−)

When both learners had finished this part, the experimenter asked them to the multi-touch tabletop and loaded the experimental setting. This consisted of a visualization of the binary questions and the answers provided by both participants (A and B, cf. Fig. 2) and the instruction to discuss the items for up to 20 min. They had the opportunity to access additional information on each item selected from the texts by pressing a blue button next to each item and were able to change the answers to the items. Additionally, in one experimental condition, dyads had information on the confidence ratings available during learning (MC+), the other one did not (MC−). After collaboration, learners were placed again in front of individual computers and individually answered the learning tasks again from scratch, including confidence ratings.

### Independent and dependent variables

Dyads were randomly assigned to one of two conditions at the beginning of the experiment: dyads receiving metacognitive confidence information during collaboration and dyads not receiving this information (between-dyad factor: MC+ vs. MC−). Additionally, we assessed our dependent variables twice within the experiment: before and after collaboration (within-subject factor: pre vs. post). Consequently, our design was a 2 × 2 factorial design with repeated measures on one factor—a common design in research on collaborative learning. Our dependent variables were the number of learning tasks correctly solved by each individual pre and post collaboration to assess knowledge gain (performance) and the number of learning tasks confidently solved by each individual pre and post collaboration to assess changes in confidence levels (confidence). Thus, while the treatment was implemented on dyad level, outcome measures were assessed for each individual separately.

### Methodological approach

Since we worked with potentially dependent data (individuals were nested within dyads), we assessed statistical interdependence between subjects within dyads with regard to our learning outcomes by computing intra-class correlation coefficients (ICC; Shrout and Fleiss 1979) for each experimental condition and each dependent variable. ICC estimates and their 95% confident intervals were calculated using SPSS statistical package version 24 based on a single-rating, absolute-agreement, one-way random-effects model (the ICC estimates are thus based on ANOVA models). While it is a common practice to calculate the ICC over the whole sample, this practice falls short for different reasons: because we assigned whole dyads to experimental conditions (between-dyad independent variable), we expect within-group variances within each condition to be lower than between-group variances (cf. hypotheses 1–2) and thus ICCs over the whole sample may partially reflect treatment effects rather than within-dyad dependencies. However, while we could partial out the treatment effect (Kenny et al. 2006), this procedure ignores possible differences in dependencies between research conditions. For example, some treatments may foster collaboration and thus interdependence between learners while others might not (cf. introduction). Since the dependencies differed between the experimental groups in our study (cf. Table 3), we decided to calculate effects based on data for each individual and repeat the analyses using dyads as units of measurement (dyad values = means over individuals within a dyad). By comparing these analyses, we get a closer look into the relationship between local dependencies and inferential as well as descriptive data, which we will describe in the results section. We added multi-level analyses for reference (cf. Table 1). We also conducted variance decompositions for the dependent variables pre and post collaboration using ANOVA models (cf. Table 4).

## Results

To test our hypotheses on learning outcomes (hypotheses 1 and 2), we conducted a two-factorial MANOVA with repeated measures on one factor. Our independent variables were experimental condition (MC+ vs. MC−) and time (pre vs. post collaboration). Our dependent variables were performance and confidence level in the learning tasks. The MANOVA was conducted once with the individual and once with the dyad as the unit of measurement. Apart from some violations of the normality assumptions for the individual data and the interdependence of the data we focus on in this paper, prerequisites were met. Since two-factorial analyses were pertinent for this design and there are no fully satisfying non-parametric alternatives, we decided to use the parametric test despite the violations. Thus, the results of the inferential statistics should be treated with caution. Level of significance was set at α = .05.

The results of the MANOVA can be viewed in Table 1. As we can see, there is a multivariate main effect of time (but not of group) and an interaction effect visible for both units of measurement. Univariate ANOVAs confirm main effects of time on both variables with performance and confidence levels rising significantly from pre to post. They also show a significant interaction effect on performance with learners in MC+ showing a steeper increase from pre to post than learners in MC− (cf. Table 2). To account for the dyadic structure, we additionally analyzed the data via a dyadic multi-level model using linear mixed modeling with restricted maximum likelihood estimation (REML) taking into account the dependencies between learners within dyads (analogous to Kenny et al. 2006), that has been used in similar studies before (e.g., Lam and Muldner 2017). While the results were similar to the other analyses, effect sizes were overall somewhat smaller and the interaction effect between time and group just missed statistical significance.

**Table 1:** Inferential statistics of the MANOVA for dyads and individuals and multilevel analyses

				Time			Group			Time × Group		
	*N*	*df*_*1*_	*df*_*2*_	*F*	*p*	η_p_^2^	*F*	*p*	η_p_^2^	*F*	*p*	η_p_^2^
Multivariate												
Dyad level	41	2	38	81.92	< .001	.81	2.04	.144	.10	3.38	.045	.15
Individual level	82	2	79	100.68	< .001	.72	2.40	.097	.06	3.55	.033	.08
Performance												
Dyad level	41	1	39	62.98	< .001	.62	0.58	.450	.01	4.63	.038	.11
Individual level	82	1	80	62.45	< .001	.44	0.64	.428	.01	4.59	.035	.05
Multilevel	82	1	39/121	52.81	< .001	.30	0.58	.450	.01	3.88	.051	.03
Confidence												
Dyad level	41	1	39	115.39	< .001	.75	2.21	.145	.05	1.90	.176	.05
Individual level	82	1	80	146.50	< .001	.65	2.78	.099	.03	2.41	.125	.03
Multilevel	82	1	39/121	109.25	< .001	.47	2.21	.145	.05	1.80	.183	.01

**Table 2:** Descriptive statistics of the results for dyad and individual data (including ICC)

	*N*		*M*	*SD*		% Decrease in *SD*	ICC
	Individual	Dyad		Individual	Dyad		
Without MC							
Performance pre	42	21	10.10	1.86	1.01	45.70	− .41
Performance post	42	21	11.50	1.76	1.62	7.95	.67
Confidence pre	42	21	11.52	2.73	2.03	25.64	.11
Confidence post	42	21	15.31	2.24	1.78	20.54	.26
With MC							
Performance pre	40	20	9.83	2.09	1.34	35.89	− .17
Performance post	40	20	12.28	1.49	1.21	18.79	.31
Confidence pre	40	20	11.20	2.13	1.57	26.29	.08
Confidence post	40	20	14.13	2.46	2.19	10.98	.56
Overall							
Performance pre	82	41	9.96	2.00	1.17	41.50	− .28
Performance post	82	41	11.88	1.67	1.47	11.98	.55
Confidence pre	82	41	11.37	2.44	1.81	25.82	.09
Confidence post	82	41	14.73	2.41	2.05	14.94	.44

### Relationship between local dependencies and inferential and descriptive statistics

We used two different units of measurement and contrasted the results due to the non-independence of the individual subjects within our sample. By violating the independency assumption due to the local dependencies within dyads, we overestimate statistical significance for individual units of measurement (by underestimating *p* values), since we assume learners within a dyad to be more similar rather than more different from random pairs. When we look at the inferential statistics for both units of measurement, we can see that *p* values increase from individual to dyad level analyses (as to be expected, since *N* is halved and is directly related to *p*). However, our effect size also increases from individual to dyad level. This is due to the elimination of within-dyad variance (by computing within-dyad means) and thus a deduction of residual variance: for individual data, we *under*estimate the within-group variance in comparison to between-group variance because of local dependence within the dyads, thereby *over*estimating the effect of the between group treatment (cf. Bliese and Hanges 2004). Replacing within-dyad variance by calculation mean scores further adds to this effect, completely evening out individual differences within dyads in the process. Thus, this procedure comes at a price: by eliminating the within-dyad variance to get rid of overestimation effects of statistical significance, we keep information about mean scores, but lose information on residual variance (cf. Table 2). Accounting for this effect by conducting a multi-level approach confirms the mostly lower effect sizes and higher *p* values.

The changes in the standard deviation due to the elimination of within-dyad variance from individual to group are presented in the %-decrease column. We also added information on ICC values and *p* (cf. Table 3). While these measures may be somewhat unstable (confidence intervals are quite large) due to the small *N* especially when looking at each experimental condition individually (Kenny et al. 1998 recommend at least 36 dyads for 80% power in detecting consequential non-independence), they still give a rough indication on within-dyad dependence. As we can see, higher ICC values are associated with a decrease in the variance lost from individual to group level. This is because learners that are more closely related do vary less between them (within dyad) than unrelated learners. Table 4 shows the decomposition of variance for each condition and outcome variable. As expected, ICC values are low for the pre-test scores, which is to be expected since the learners within each dyad did not collaborate yet in any way (please note that learners within a dyad received different versions of the learning text, leading to negative ICC values for performance pre collaboration—since negative values violate the ICCs model assumption, associated *p* values may not be interpreted). Variance decomposition shows that dyads do not account for much of the variance pre collaboration and error variance is quite high. Thus, computing a mean dyad score of largely unrelated learners eliminates large amounts of variance in pre-test. However, as dependencies (and thereby ICC values) increase in post-test due to collaboration and a large amount of the variance can be explained by dyads, the loss in variance decreases. Meanwhile, the means stay identical since the dyad value was computed as a mean between dyad members. It is interesting to notice that within the MC− condition, learners’ performance scores are more closely related to each other than in the MC+ condition (cf. ICC in Table 3), and thus, losses in variance (and information) are greater for the latter condition, if we combine the data to get dyad level data (cf. Table 2).

**Table 3:** Intra-class correlation coefficients per group and outcome variable

	Intra-class correlation coefficient (ICC)	95% Confidence interval		*p*
		Lower bound	Upper bound	
Without MC				
Performance pre	− .41	− .70	.02	.971
Performance post	.67	.36	.85	< .001
Confidence pre	.11	− .32	.51	.308
Confidence post	.26	− .18	.61	.120
With MC				
Performance pre	− .17	− .56	.28	.772
Performance post	.31	− .13	.66	.079
Confidence pre	.08	− .35	.50	.355
Confidence post	.56	.17	.80	.004
Overall				
Performance pre	− .28	− .54	.02	.966
Performance post	.55	.29	.73	< .001
Confidence pre	.09	− .22	.39	.277
Confidence post	.44	.16	.66	.001

**Table 4:** Variance decomposition (ANOVA) for performance and confidence pre and post collaboration

	Var(dyad)	Var(error)	Var(overall)	Var(dyad)	Var(error)	Var(overall)
	Performance pre			Performance post		
Without MC	− 1.39	4.81	3.45	2.11	1.02	3.09
With MC	− 0.74	5.08	4.35	0.70	1.53	2.20
	Confidence pre			Confidence post		
Without MC	0.83	6.62	7.43	1.29	3.74	5.00
With MC	0.38	4.15	4.52	3.42	2.73	6.06

If we look more closely at the actual variance, we can see that in the MC− condition, variance for individual performance data is more or less equal in pre and post (slight decrease), whereas on dyad level, variance noticeably increases (cf. Table 2). Factoring in the high interdependence in post-test for this group, the data suggests that while learners within dyads may be more similar post collaboration, the dyads themselves seem to grow apart. This is especially interesting since we do not see the same effect for MC+: here, the variance on the individual level decreases visibly from pre to post, while the decrease on dyad level is negligible. Table 4 shows that while error variance is roughly similar between the conditions for performance post, the variance explained by the dyad is three times higher for learners without metacognitive information (MC−). For confidence, we get a different picture: although there are no overall effects of the treatment (cf. Table 1), the ICC after collaboration is much higher for learners within MC+ (cf. Table 3). Thus, while the loss in variance from individual to dyad level is rather similar pre collaboration, the losses are visibly smaller post collaboration within MC+ (cf. Table 2). Table 4 supports this notion: we can see that while unexplained variance for confidence post collaboration is smaller in MC+, the variance explained by the dyad is more than twice the size as in the MC− condition. From pre to post however, overall variance decreases in the MC− condition and increases in the MC+ condition (both units of measurement). With a larger percentage of variance in post being due to between dyad variance for MC+ than for MC−, on a dyad level, the difference between the groups is more obvious than on an individual level.

## Discussion

Our experimental study aimed at investigating whether metacognitive confidence information may be a valuable contribution to information on specific assumptions in group awareness tools. As argued before, adding metacognitive information (i.e., subjective evaluations on one’s knowledge) may be used to validate assumptions and thus foster grounding processes (Clark and Brennan 1991), enabling learners to better tail their learning processes to each other (Clark and Murphy 1982), leading to better learning (Dehler et al. 2011). Our inferential analyses with regard to our hypotheses coherently indicate that learners gain knowledge and confidence during collaboration, but the treatment does not affect confidence gain (hypothesis 2). For performance gain (hypothesis 1), the effects are also similar for all analyses, and—taken together—the evidence gently points toward a possible treatment effect, although multi-level analyses just missed the level of statistical significance. Without more specific analyses of the interdependencies, we might thus have cautiously concluded that adding metacognitive information may foster collaboration processes relevant for learning. However, looking at the interdependencies, it is startling that learners without the metacognitive information are higher interdependent regarding their performance, while still performing somewhat worse. Thus, interpreting that the treatment does foster collaboration processes seems to fall short. One more suitable explanation might be that learners without metacognitive information more explicitly target differences between them rather than discussing the underlying concepts needed to gain knowledge. Such an approach might tail in with quick consensus building (cf. Weinberger and Fischer 2006) and may account for both the high interdependence as well as the somewhat lower knowledge gain for this condition. While it seems that learners with metacognitive information available collaborated differently, we would have expected improved collaborative learning processes to lead to more aligned performances within dyads as well. However, we did not observe such an alignment. This might be explained by the value learners assign to low confidence assumptions. Confidence cues are used to judge the knowledge of others, but also the validity of assumptions (e.g., Price and Stone 2004), and confidence in assumptions is also seen as a prerequisite for experiencing cognitive conflict (Lee et al. 2003). If confidence information is visualized and thus more salient, in cases of low confidence this might reduce the learners’ need to align their understanding and reach consensus about the content. As discussed before, while aligned performance levels do not necessarily come with conversion of assumptions, differences in basic assumptions could explain some of the differences and may explain why dyads do not explain a lot of the variance within the condition with metacognitive information. Alternatively, providing metacognitive confidence information may also have made learners assume different roles within the process according to their respective confidence levels (e.g., more confident student mainly explaining and providing information), which may have led to less reciprocal yet still effective learning processes. In such a scenario, the dyad itself might still explain some variance, but less, and the variance within dyads (error variance) should be comparably high. While confirming any of these interpretations would of course require further research, the different levels of local dependencies cast doubt about the assumption that metacognitive information simply enhanced and guided reciprocal knowledge exchange processes. On a descriptive level, the variances for dyad level data on performance show that dyads without metacognitive information become more diverse from pre to post while dyads with the information rather become more similar, so that most of the variance is due to individual error. One plausible explanation would be that dyads with metacognitive information use the provided information in a similar fashion resulting in similar mean performance, while dyads without such information apply slightly more diverse approaches. While this may explain the differences in variance due to dyad, it does not explain what strategies may lead to higher within-dyad variance (error variance) and lower between-dyad variance, except for more individualistic approaches and less collaboration.

As for confidence, overall results did not show any differences between the conditions neither on dyad, on individual, nor on multi-level. Interestingly, although performance scores seem to be more related for learners without metacognitive information, confidence scores are more interdependent for learners with metacognitive information available. This may be due to the fact that learners with metacognitive information actively align their confidence levels, but ultimately without gaining more or less confidence in the process. On the dyadic level, we see slightly lower variance for the condition without metacognitive information and thus, the dyads rather than the individuals seem to be more alike. Thus, dyads may have different approaches to learning that affect confidence levels differently if metacognitive information is provided, leading to greater variance between dyads, but interrelated approaches within. Further research should look into those approaches as they may not only account for interindividual differences but also explain why there was no overall effect on confidence levels while the differing ICC values indicate that the treatment had some effect on the collaboration process relevant to confidence levels. An alternative explanation may be re-interpretations of own knowledge in light of social information as has been found for example with regard to information on performance of others (Fraundorf and Benjamin 2016) or on co-learners having questions about the material (Karabenick 1996). In our study, learners may have established an agreement on how overly confident they were about the learning material, especially when confidence information was provided. While negotiating agreement is a collaborative act, this explanation focuses more on the common exposure aspect of interdependence, because such alignment processes may well happen without the learners interacting or explicitly discussing confidence if metacognitive information is provided. Finally, it is important to point out that unexplained (error) variances in confidence levels were quite different between the conditions pre collaboration and thus, some effects might be due to random differences between the learners rather than experimental treatment.

Of course, our descriptive analyses of changes and differences in variances are not suitable to draw definite conclusions. Rather, they provide clues into possible mechanisms of collaboration that may be used to generate hypotheses to be tested in further studies. Similarly, while the ICC values post collaboration seem very different descriptively, confidence intervals are quite large due to the small samples and considerably overlap, so jumping to conclusions may be premature. However, they still provide however-fragile evidence that suggests that collaboration processes might have been affected by the treatment in an unexpected way and should thus be further examined.

Overall, comparing dyadic and individual level data showed that both approaches produced similar outcomes for our data. Multi-level analyses reached similar conclusions, although the effect sizes were smaller and the interaction effect on performance was not statistically significant. Since the advantages of multi-level approaches to analyze data within collaborative learning settings have been repeatedly illustrated elsewhere (for detailed examples contrasting results drawn from individual, dyadic, and multi-level analyses, see, e.g., Janssen et al. 2011), we did not compare multi-level results with dyadic and individual data in detail. Rather, we argue that looking into the ICCs and variances allowed us to gain some insight into the collaboration processes. Using this additional information, we conclude that providing confidence information may have led learners to focus on different aspects of the collaboration—aligning confidence rather than performance levels. While this may lead to higher performance gains, the mechanisms need to be further investigated. To achieve this, methodological one-track approaches are insufficient. Causal models require a sound description of generative mechanisms able to explain variations within the data and quantitative methods are limited to testing for differences and co-variations. Thus, analyses of variance have limited capacity to help us understand the underlying processes of the observed phenomena as they reduce social reality to a fixed set of linear relationships largely disregarding social-contextual complexity and dynamics (cf. Abbott 1988). A meaningful integration of theoretical and statistical models thus requires a combination of in-depth analyses of collaborative and transactive processes to explain observed variances within the data by providing a rationale of causal relationships (e.g., via qualitative analyses of the interaction and communication processes) and inferential statistics to secure these findings on a larger scale.

## Conclusion

Local dependencies in collaborative research are often unwelcome in light of constrains they put on statistical analyses. However, it is important to keep in mind that such dependencies may result from favorable interaction processes. Collaborative learning scenarios often explicitly target such processes of learners exchanging information, co-constructing knowledge, or in other ways interacting while influencing the learning partners’ cognitive processes (Dillenbourg 1999). Hence, the focus of our study was to exemplarily show how interdependencies (i.e., ICCs) may be used to gain more insight into the mechanisms of collaboration. Comparing results of statistical analyses between different units of measurement (individual vs. dyad) and decomposing variance may further provide valuable information easily lost when compensating for these effects rather than interpreting them. While none of the statistical aspects of local dependence discussed in this paper are genuinely new, the results described demonstrate the effect this has on specific dyadic data drawn from a study typical for quantitatively assessing the effect of a treatment to foster collaboration on individual learning outcomes. We argue that this valid information should not be viewed as hampering our statistical design, but as enriching our analyses by providing valuable information. As stated before, interdependence is not a mere statistical phenomenon, but needs to be interpreted psychologically as the result of collaborative processes or shared experiences (Cress 2008). Thus, it has theoretical value and should be critically analyzed, especially if the research conducted targets the aforementioned collaboration processes as frequently done in CL research. Interventions designed to support collaborative learning processes affect the interaction processes between learners. These processes are pivotal to collaborative learning, where peers interact while pursuing a learning goal (Dillenbourg et al. 2009; Suthers 2012) and thus, it is reasonable to assume that such interventions affect the interdependence between learners. While in cooperative settings this might be somewhat different (when learners split their work and focus on very different aspects of a task), we argue that when considering collaboration within CL, our underlying theoretical assumptions about collaborative processes should often lead us to expect interdependencies and their absence might be a reason to rethink these assumptions. Apart from adjusting statistical models to the characteristics of our specific data, using quantitative and qualitative methods to take a closer look at the fit between our model assumptions about collaborative processes taking place in a specific educational scenario and the outcome data retrieved is a great opportunity to adjust our assumptions about educational practices and ultimately build better models.

## Abbreviations

CL: Collaborative learning
CSCL: Computer-supported collaborative learning
ICC: Intra-class correlation coefficient
MC: Metacognitive confidence information
MC−: Research condition without information on the learners’ metacognitive confidence
MC+: Research condition with information on the learners’ metacognitive confidence
